# iTRAQ-based quantitative proteomic analysis in vernalization-treated faba bean (*Vicia faba* L.)

**DOI:** 10.1371/journal.pone.0187436

**Published:** 2017-11-09

**Authors:** Yun-Ying Cao, Xiao-Chun Bian, Mo-Xian Chen, Li-Ru Xia, Jianhua Zhang, Fu-Yuan Zhu, Chun-Fang Wu

**Affiliations:** 1 Jiangsu Yanjiang Institute of Agricultural Sciences, Nantong, China; 2 College of Life Sciences, Nantong University, Nantong, Jiangsu, China; 3 College of Biology and the Environment, Nanjing Forestry University, Nanjing, Jiangsu Provinve, China; 4 Shenzhen Research Institute, The Chinese University of Hong Kong, Shenzhen, China; 5 Jiangsu Academy of Agricultural Sciences, Nanjing, China; 6 Department of Biology, Hong Kong Baptist University, and State Key Laboratory of Agrobiotechnology, The Chinese University of Hong Kong, Shatin, Hong Kong; Northeast Forestry University, CHINA

## Abstract

Vernalization is classically defined as the induction of flowering process by exposure of the plants to a prolonged cold condition. Normally, it is considered as a precondition of flowering. *Vicia faba*, commonly known as faba bean, belongs to family *Fabaceae*. It is one of the plant species that has been cultivated in the earliest human settlements. In this study, an iTRAQ-LC-MS/MS-based quantitative proteomic analysis has been conducted to compare the vernalized faba bean seedlings and its corresponding control. In total, 91 proteins from various functional categories were observed to be differentially accumulated in vernalized faba bean seedlings. Subsequent gene ontology analysis indicated that several biological processes or metabolic pathways including photosynthesis and phytic acid metabolism were differentially respond to vernalization in comparison to the control sample. Further investigation revealed that a family of proteins nominated as glycine-rich RNA-binding factor was accumulated in vernalized seedlings, indicating an extra layer of regulation by alternative splicing on transcript abundance in response to vernalization. These findings raise a possibility that these candidate proteins could be important to represent the responsive network under vernalization process. Therefore, we propose that the regulation of vernalization in faba bean not only occurs at the transcriptional level as previously reported, but also at the post-transcriptional level.

## Introduction

Faba bean (*Vicia faba* L.) is a common legume belonging to an annual or biennial herb [1]. It originated in the central and western of Asia and has become the largest consumption of beans in China [2]. Faba beans are widely cultivated in the world due to their rich nutrients for food and their multiple agronomic benefits including the improvement of soil sanitation, soil structure as well as the moisture retention [3], which was received increasing attention in legume crops. Meanwhile, faba bean was also a winter-sown bean in the southern of china, which can fully take advantage of larger numbers of idle arable lands and labor resources in winter, leading to increase the income of farmers and develop the agricultural economy as well as improving the soil environment.

Plant flowering is mediated by both endogenous hormone signals and exogenous environmental cues including photoperiod and temperature [4]. Low-temperature stress is a critical factor to limit crop production. However, nonfreezing temperatures during the vernalization range with a long time exposure could greatly accelerate flowering in winter annual crops such as wheat, barley, cabbage and faba bean [5,6]. Vernalization plays an important role in regulation of flowering time in higher plants for flower transformation, which is an evolutionarily adaptive mechanism preventing the plant growth transition from the vegetative to the reproductive phase before winter and allowing flowering in the favorable conditions of spring [6,7]. The molecular basis of vernalization in plants is to repress flowering by down-regulating flowering-related genes. In Arabidopsis, the effect of vernalization epigenetically modify the chromation structure belong to a clade of strong flowering repressors such as *FLOWERING LOCUS C* (FLC), from its active state to its silenced one, via histone modification [8,9]. In cereals, three vernalization response loci nominated as *VRN1*, *VRN2* and *VRN3* have been identified by genetic studies comparing winter and spring cultivars of wheat and barley [8,10,11]. Similar flowering repressing mechanism in the vernalization circuitry was observed between Arabidopsis and cereals. Interestingly, a positive feedback loop created by the dual role of VRN1 constitutes the new vernalization componenet in cereals which was not found in Arabidopsis [8], indicating the respective vernalization pathways between Arabidopsis and cereals evolved independently. However, the vernalization process has been thoroughly studied in *Arabidopsis* and wheat during the past years but the molecular regulatory mechanisms of vernalization in other plants including cabbage, maize and faba bean are still largely unknown. Due to the striking differences between the circuitry and components of vernalization in Arabidopsis and Wheat, it is therefore necessary to perform more extensive investigations to further explore and dissect the range of vernalization mechanisms that exist in more flowering plants, particularly the cereals possessing agronomic benefits such as faba bean described above.

A proteomics approach proved to be a powerful methodology to characterize plant responses to different biotic and abiotic stresses [12], which has been recently applied into investigate the proteome changes under vernalization or chilling treatment in several crop species including rice, maize and wheat[13–15]. However, the proteomics analysis of faba bean after vernalization is rarely reported. In this study, the changes in proteome profile in faba bean subject to the vernalization were examined using the Isobaric Tag for Relative and Absolute Quantification (iTRAQ) LC-MS/MS approach, which can provide more accurate quantification of differentially expressed proteins and large-scale identification of vernalization-related proteins comparing with the traditional 2D-gel based proteomic approach [16]. A total of 2766 proteins were quantitatively identified in seedlings of faba bean (*Vicia faba L*.), 91 of which were found to be differentially expressed after vernalization (cut-off: ratio 1.5 or 0.67 at *P* < 0.05). Following detailed data analysis revealed a down accumulation of several photosynthesis-related proteins and up-regulation of phytic acid biosynthesis pathway as well as a clade of glycine-rich RNA-binding proteins potentially critical for the vernalization response. Substantial information on the vernalization-responsive proteome changes was also acquired, offering deeper understanding to elucidate and explore the potential molecular mechanism for the adaptive ability of faba bean to the vernalization effects.

## Materials and methods

### Plant materials and treatment conditions

Faba bean (*Vicia Faba* L.) cv. Tongxian 2 were grown in a greenhouse at a 12-h day/12-h night cycle, at 20–25°C. At the age of two weeks, the treatment group was transferred to 0–4°C for 20 days. Stems of treatment group and blank control group were obtained for protein extraction.

### Antioxidant enzymes assay

Leaf and shoot tips were first sampled before treatment, and successively sampled at 6, 12, 18, 24 and 30 d after the treatment. The antioxidant enzyme activities including peroxidase (POD), catalase (CAT) and superoxide dismutase (SOD) have then been determined according to Wang et al.^17^ with minor modifications.

In brief, 0.2 g mass of fresh sample (FM) was homogenized in the extraction buffer consisting of 50 mM sodium phosphate buffer, pH 7.8 and the supernatant for measuring POD and CAT enzyme activities. POD activities were assayed by the oxidation of guaiacol in the presence of H_2_O_2_. The increase in absorbance was recorded at 470 nm. The reaction mixture of 4 ml containing 1 ml 50 mM phosphate buffer (pH 7.0), 1.95 ml 0.2% (w/v) H_2_O_2_, 0.95 ml 0.2% (w/v) guaiacol and 0.1ml enzyme extract. CAT activity was assayed by measuring the decrease in absorbance at 240 nm in a 3 ml reaction mixture consisting of 1.9 ml dd H_2_O, 1.0 ml 0.2% (w/v) H_2_O_2_ and 0.1 ml of enzyme extract. The increase or decrease of 0.01 OD value per min was defined as one Unit (U) activity of POD and CAT. The POD or CAT activity was thus expressed as U mg^–1^(protein) min^–1^.

For SOD activity determination, approximately 0.5 g fresh sample was homogenized in the extraction buffer consisting of 50 mM sodium phosphate buffer, pH 7.8, 0.1mM ethylene diamine tetraacetic acid (EDTA), 0.3% (w/v) Triton X-100 and 4% (w/v) polyvinyl polypyrrolidone for determination SOD activity. The assay mixture of 3 ml contained 14.5 mM L-methionine 2.7 ml, 3.0 μM EDTA 33.3μl, 2.25 mM nitroblue tetrazolium (NBT) 33.3μl, 60 μM riboflavin 33.3μl and 10~50μl enzyme extract. The photoreduction of NBT (formation of purple formazan) was measured at 560 nm. One unit of SOD activity was defined as extract volume that caused 50% inhibition of the photoreduction of NBT and expressed in U g^–1^(FM) h^–1^. Each result of SOD, POD and CAT was the mean of three replications.

### SPAD measurement

All samples were measured the values of SPAD (SPAD-502 type, Konica Minolta, INC. Japan) at 0, 6, 12, 18, 24 and 30 days after treatment. For every treatment, ten plants were selected for the analysis. Top three functional leaves from every plant were used for measurement. Every time point was measured five times to calculated the average values.

### Protein extraction

The vernalization-treated and untreated plants (approximately 1g) were ground in liquid nitrogen and homogenized with lysis buffer (8 M urea, 2 mM EDTA, 10 mM DTT and 1% Protease Inhibitor Cocktail). The samples were centrifuged by 20000g for 10 min at 4°C. Subsequently, the supernatants were acquired for protein precipitation with chilled 15% TCA for 2 h at -20°C. After centrifugation and removing supernatant, the pellets were washed with chilled acetone for three times. Finally, the proteins were dissolved in the buffer (8 M urea, 100 mM TEAB, pH 8.0). Protein concentrations were determined by the 2-D Quant kit. Three biological replicates were prepared from vernalization-treated and untreated plants.

For digestion, the protein samples (100 μg each) were reduced by 10 mM DTT at 37°C for 1 h with gentle shaking. Reduced samples were alkylated by 20 mM iodocaetamide (IAA) for 45 min in darkness. Subsequently, they were then further diluted by adding 100 mM TEAB for the urea concentration below 2M. Finally, trypsin digestion was conducted (1:50 w/w enzyme/protein) overnight at 37°C, followed by a second digestion (1:100 w/w enzyme/protein) for 4 hours.

### iTRAQ labeling and strong cation exchange (SCX) chromatography

After trypsin digestion, peptides were desalted by the Strata X C18 SPE column (Phenomenex) and vacuum evaporation. Peptides were reconstituted in 0.5 M TEAB and treated using 6-plex TMT kit according to the manufacturer’s instructions. Briefly, Desalted peptides from untreated samples including three biological replicates were labeled by the iTRAQ tags 129, 130 and 131, whereas peptides from vernalization-treated samples were labeled with the iTRAQ tags 126, 127 and 128. Subsequently, the sufficient peptide mixtures harboring different tags were pooled, desalted and evaporated by the vacuum centrifugation. The samples were then fractionated using Agilent 300 Extend C18 column (5 μm particles, 4.6 mm ID, 250 mm length) by the strong cation exchange (SCX) chromatography. The eluted-peptides were then combined into 18 fractions and vacuum-dried.

### Peptides analysis by LC-MS/MS

Q Exactive^™^ plus hybrid quadrupole-Orbitrap mass spectrometer (ThermoFisher Scientific) coupled to an EASY-nLC 1000 UPLC system equipped with a reversed-phase analytical column (Acclaim PepMap RSLC, Thermo Scientific) for peptides analysis. Peptides were separated by a ladder gradient from 6%-36% of solvent B (0.1% formic acid in 98% acetonitrile) including 6%-10% for 4min, 10%-23% for 22min, 23%-36% for 8min with a constant flow rate of 300nl/min. The acquired peptides were analyzed by the Orbitrap with an electrospray voltage of 2000V. Data-dependent acquisition was conducted by a survey scan of 250ms in the range 350 to1800 m/z for the collection of MS1 spectra. The top 20 precursor ions were selected for the MS/MS fragmentation with 30s dynamic exclusion.

Peptides were dissolved in 0.1% FA, directly loaded onto a reversed-phase pre-column (Acclaim PepMap 100, Thermo Scientific). Peptide separation was performed using a reversed-phase analytical column (Acclaim PepMap RSLC, Thermo Scientific). The gradient was comprised of an increase from 6% to 10% solvent B (0.1% FA in 98% ACN) over 4 min, 10% to 23% in 22 min, 23% to36% in 8 min and climbing to 85% in 5 min then holding at 85% for the last 3 min, all at a constant flow rate of 300 nl/min on an EASY-nLC 1000 UPLC system, The resulting peptides were analyzed by Q Exactive^™^ plus hybrid quadrupole-Orbitrap mass spectrometer (ThermoFisher Scientific).

Mascot search engine (v.2.3.0) was used to search all of the MS/MS data thoroughly against the *vicia* protein database. Mass errors for the precursor and fragment ions were set as 0.05 and 0.02, respectively. Trypsin digestion and cysteine alkylation were specified as parameters in the database searching. For protein quantification method, TMT-6-plex was selected in Mascot. A global false discovery rate (FDR) of < 1% was used and peptide ion score of ≥ 20 was preset. The full identification and quantification of protein list was shown in S2 Table.

### Bioinformatics analysis

Gene Ontology annotation (GOA) database (http://www.ebi.ac.uk/GOA/) and InterPro platform (http://www.ebi.ac.uk/interpro/) were coordinately used for the proteins functional annotation. The functional classification based on three ontologies including biological process, cellular component and molecular function was performed by the Gene Ontology annotation (http://www.geneontology.org/). Protein pathway analysis was commonly conducted by the Kyoto Encyclopedia of Genes and Genomes (KEGG) (http://www.genome.jp/kegg/) database. Meanwhile, the subcellular localization predication of proteins was used by wolfpsort (http://wolfpsort.seq.cbrc.jp/) software. For the cluster analysis, the quantified proteins in this study were divided into four quantitative categories according to the quantification ratio to generated four quantitative categories: Q1 (0< Ratio <1/1.5), Q2 (1/1.5 < = Ratio <1/1.3), Q3 (1.3< Ratio < = 1.5) and Q4 (Ratio >1.5). Then, the quantitative category based clustering was performed by the heat map as previously described.

### Quantitative real-time RT-PCR analysis

Transcriptional investigation of genes corresponding to vernalization-responsive proteins in faba bean was conducted by quantitative real-time RT-PCR. RNA samples were prepared from faba bean seedlings after 18 days of vernalization treatment. Total RNA was reverse-transcribed into cDNA using M-MLV reverse transcriptase (Promega) according to the manufacturer’s instruction. The SYBR Green Mix (Applied Biosystems) was used for the qRT-PCR analysis and the PCR was conducted on a StepOne Plus realtime PCR system under the optimized program as followings: 95°C for 5 min followed by 38 cycles of 95°C for 15 sec and 58°C for 45 sec. Fold changes in expression level was calculated by the comparative CT value method [17].

## Results

### Sampling time evluation for vernalization fulfillment by the physiological characterization

**Fig 1:**
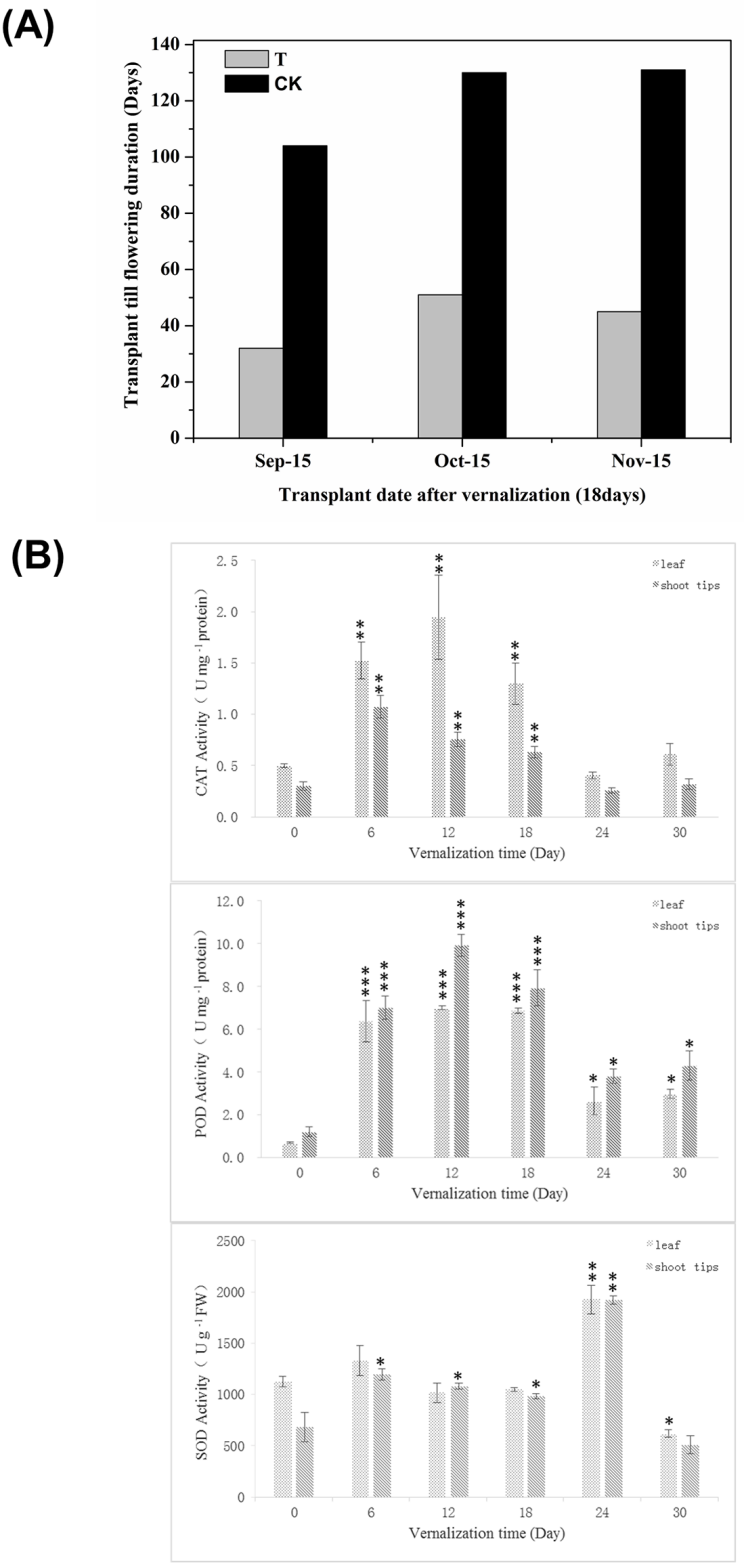
Physiological characterization of vernalization-treated (T) and non-treated (CK) faba bean seedlings. Analysis of antioxidant enzyme activities was performed in Faba bean seedlings of leaf and shoot tips during different vernalization days. (A) CAT, (B) POD, (C) SOD. Values expressed are means ± *SD* of three replicates. Student’s *T* test was conducted on the values between different vernalization time and non-vernalization treatment. *P<0.05; **P<0.01; *** *P*<0.001 by Student’s *t* test.

The appropriate time to vernalization fulfillment is essential for our proteomic investigation on the vernalization-treated faba bean. The representative biological significance of vernalization was able to influence the flowering time in plants [18]. Obviously, Around 80 days advance of early flowering was observed in vernalization-treated faba bean seedlings compared to the non-treated faba bean seedlings (Fig 1A), which confirm the effectiveness of vernalization treatment in Faba bean. Furthermore, besides the shoot growth development variation under different vernalization time, the endogenous antioxidant activity after vernalization process was also considered as one of important physiological parameter to determine the vernalization fulfillment in faba bean [19]. Therefore, the antioxidant enzymes including CAT, POD as well as SOD assays from leaf and shoot tips were performed under different vernalization treatment time. As shown in Fig 1B, both CAT and POD activity exhibited a growing increasing subject to 18 days of vernalization treatment, followed with a gradually decreasing after 18 days till 30 days, while SOD activity reach a peak at 24 days in compared with the nonvenrnalization-treated faba bean (Fig 1B). Thus, the vernalization fulfillment range of faba bean should be 18 days to 24 days based on this antioxidant enzymes assay and the vernalization treatment for 18 days was used in this proteome study.

**Fig 2:**
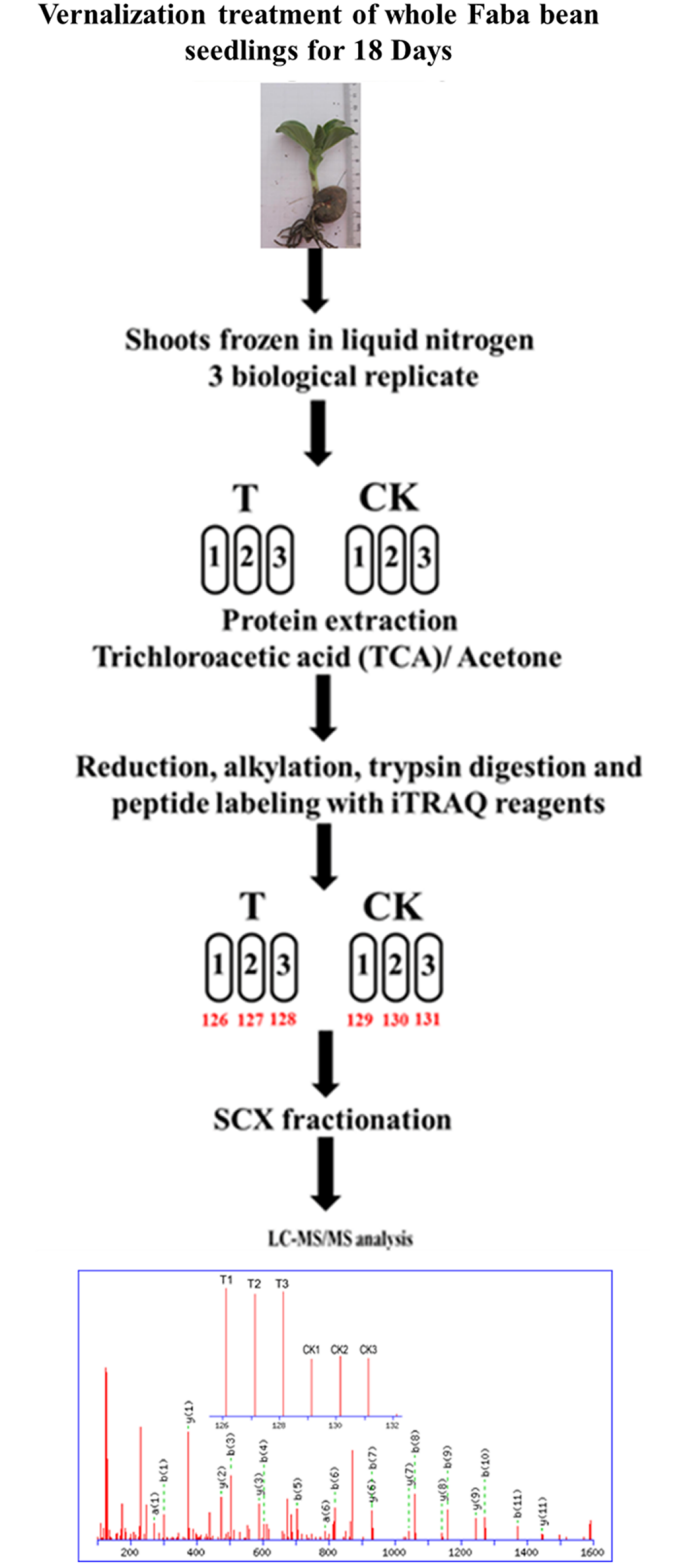
Workflow for the iTRAQ-based quantitative proteomic experiment. Protein samples were obtained from vernalization-treated (T) and non-treated (CK) faba bean seedlings. Three biological replicates of T and CK samples were digested and labeled with iTRAQ tags, followed by the SCX fractionation. The sufficient amounts of labeled peptides were used for LC-MS/MS analysis.

To understand the effects of vernalization on proteome changes in faba bean seedlings, iTRAQ-based proteomics investigation was performed. The samples were labeled with six iTRAQ tags (T-1:126, T-2:127, T-3:128; CK-1:129, CK-2:130, CK-3:131) and pooled for fractionation by SCX chromatography. A total of 12 selected fractions containing sufficient amounts of labeled peptides were used for LC-MS/MS analysis. The schematic workflow of the above experimental procedures is shown in Fig 2.

### Global characterization of proteome changes between vernalization-treated (T) and nonvernalization-treated (CK) faba bean seedlings

To obtain a comprehensive observation on faba bean responses to the vernalization, comparative proteomic analysis was performed. After merging data from three biological replicates, a total of 4172 proteins were identified, among which 2766 proteins were quantified. The distribution of mass error and length of all the identified peptides as well as the repeatability of the replicates were also provided (S1 and S2 Figs). The length of most peptides enriched on 8 to 16 with the mass error below 0.02 and a high performance of pearson correlation coefficient in repeated samples, indicating a high quality of the MS data and sample preparation in this iTRAQ-based experiment. For the vernalization-responsive proteins, only those showing a fold change of above 1.5 or below 1/1.5 (*P* < 0.05) in the quantitative ratios were considered. We totally identified 91 proteins showed significant changes in protein abundances with 29 up-regulated and 62 down-regulated proteins (S1 Table).

**Fig 3:**
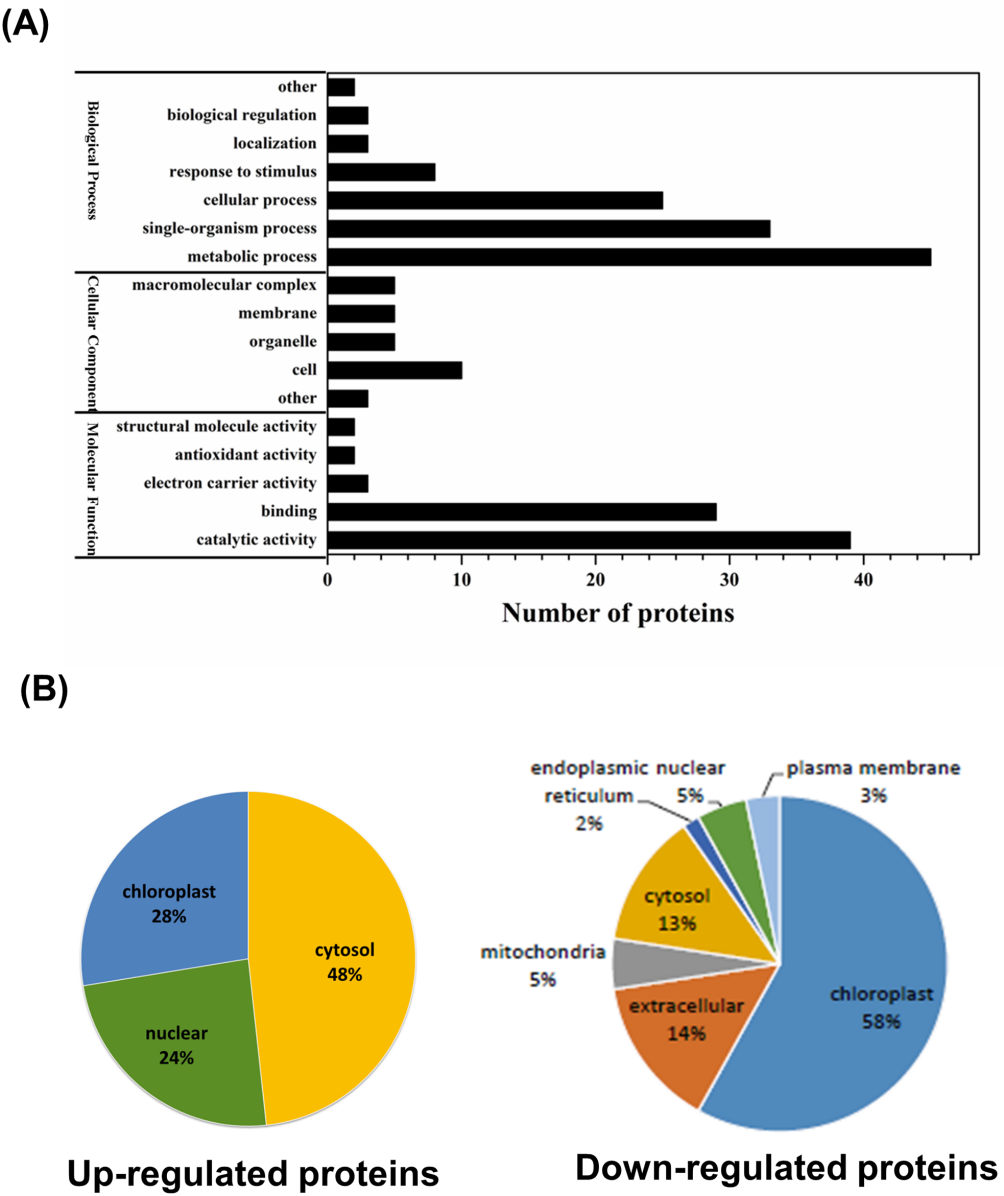
Gene Ontology (GO) analysis in the vernalization-responsive proteins. (A) Plots reveal the GO distribution of differentially expressed proteins identified in the vernalization-responsive proteins in Faba bean. (B) Subcellular location analysis of up- and down-regulated proteins showing the distribution in cellular component.

Those vernalization-responsive proteins were further assigned to several different categories including Gene Ontology, Subcellular Localization and Cluster analysis (Fig 3). Gene Ontology (GO) analysis were conducted by three main ontologies including biological process, cellular component as well as molecular function, among which biological processes accounted for 7 GO terms, cellular component accounted for 5 GO terms, and molecular function accounted for 5 terms. Functional GO enrichment analysis revealed that most of the identified vernalization-responsive proteins concentrated into the metabolic processes with the feature of RNA-binding or catalytic activity (Fig 3A). Subcellular localization analysis also revealed that most of up-regulated proteins locate into cytosol whereas the down-regulated proteins were enriched in the chloroplasts (Fig 3B), suggested that the specific cellular localization and biochemistry characteristics among the differentially expressed proteins highly correlated with the vernalization effects in Faba bean. In addition, the quantified proteins in this study were further divided into four quantitative categories for the comprehensive evaluation on the identified proteins by cluster analysis. According to the quantification ratio to generate four quantitative categories: Q1 (0<Ratio<1/1.5), Q2 (1/1.5< = Ratio<1/1.3), Q3 (1.3<Ratio< = 1.5) and Q4 (Ratio>1.5), the detailed cluster analysis based on the GO enrichment, Protein domain as well as KEGG pathway enrichment were reported as shown in S3 and S4 Figs, which provide a hierarchical view on the most significant proteome changes in faba bean after vernalization.

### Photosynthesis-related proteins are down accumulated after vernalization

**Fig 4:**
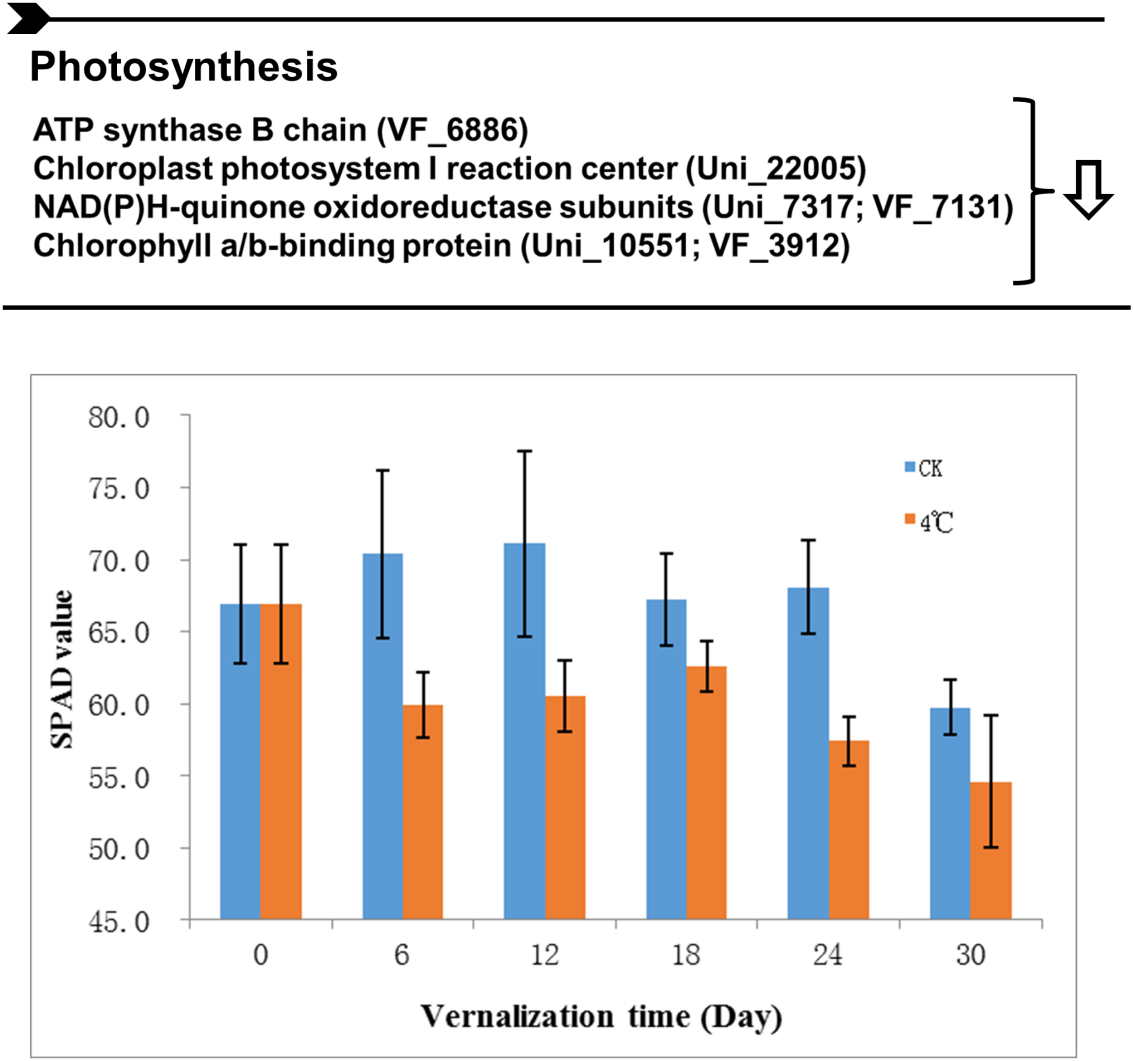
The down accumulation of photosynthesis-related proteins and metabolic process after vernalization. List of photosynthesis proteins exhibited decreased abundances in response to vernalization in Faba bean seedlings. Analysis of SPAD in faba bean after different vernalization time was also performed. Values expressed are means ± *SD* of three replicates.

Low temperatures can greatly inhibit the photosynthesis through the mediation of the photo-oxidation [20]. Although photochemical processes such as the light energy trapping by the photosystems are temperature independent, the redox reactions associated with electron transport chain are temperatures dependent leading to an energy imbalance upon low temperature [13,20]. Accordingly, our quantitative proteomic analysis announced all the six differentially expressed photosynthesis-related proteins exhibited reduced abundance by approximately 30–40% after vernalization treatment (S1 Table). Those proteins are main components of photosynthetic enzymes complexes including photosystem (PSI), photosynthetic electron transport and ATP synthase (Fig 4), suggesting that the photosynthetic functions were strongly impaired. For example, one core protein of PSI-PSII complex (Uni_22005), two in electron transport (Uni_7317 and VF_7131) as well as an ATP synthases (VF_6886) are important subunits involving the photosynthesis process, which contribute together to the transformation from light energy to chemical energy in plants [21]. The down accumulation of chlorophyll a-b binding proteins including Uni_1055 and VF_3912 besides affect chlorophyll synthesis in plants [22], may also alleviate the photo-damage due to the elevation of ROS [14]. Consistently, the decreased chlorophyll contents changes were also observed in faba bean under different vernalization days (Fig 4), further suggested that the retardation of photosynthesis was one kind of conserved vernalization effect in plants. Taken together, the overall down-regulation of photosynthetic metabolism in faba bean seedling after vernalization possibly induced an exclusive environment, which influences the activity of downstream enzymes involving the glycolysis and sucrose metabolism, thus adversely affect the energy balance and flowering process in plants.

### Phytic acid biosynthesis pathway is induced by the vernalziation treatment

**Fig 5:**
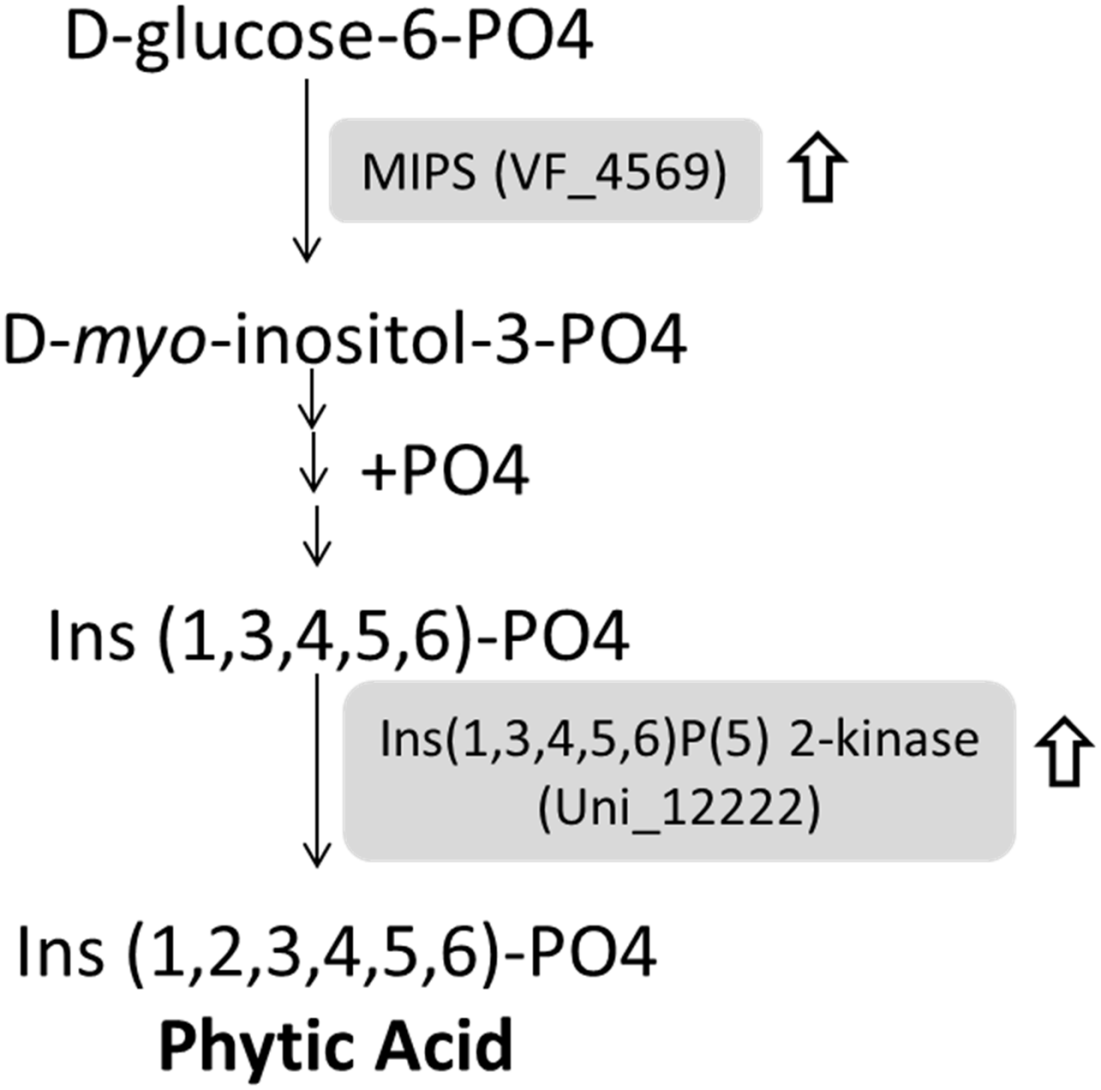
Up-regulation of phytic acid biosynthesis pathway upon vernalization treatment. Two enzymes involved in phytic acid biosynthesis are up-regulated (indicated by up-arrow symbol). MIPS (VF_4569) is responsible for the conversion of D-glucose-6-PO4 to D-myo-inositol-3-PO4, and Ins(1,3,4,5,6)P(5) 2-kinase (Uni_12222) participate in the sequential phosphorylation for the biosynthesis of phytic acid.

The phytic acid biosynthesis pathway was highly represented in the vernalization-treated proteome and several enzymes involved in phytic acid biosynthesis showed increased abundance upon vernalization treatment, suggesting a potential role for phytic acid in faba bean in response to vernlization (S1 Table and Fig 5). For example, MIPS (VF_4569) catalyses the conversion from D-glucose-6-PO4 into D-*myo*-inositol-3-PO4, which is the crucial product essential for the biosynthesis of inositol and phytic acid [23]. Such *myo*-inositol possess multiple functions and involved in many important biological processes including stresses responses, developmental growth regulation as well as signal transduction [24]. Subsequently, D-*myo*-inositol-3-PO4 is directly transformed to phytic acid through the sequential phosphorylation by a clade of kinases including Ins (1,3,4,5,6)P(5) 2-kinase (Uni_12222) (Fig 5). The phytic acid is mainly accumulated in legumes grains, tubers, pollens, which contains an inositol ring accompany with six phosphate groups and consider as the main and the most stable storage form of phosphorus in plants [25]. The reduction of MIPS activity and phytic acid contents in cereals mutant including rice, maize, potato, barley and soybean causes severe restriction of plant growth, indicating their essential role in plant physiology and growth development[26–29]. For an instance, transgenic potato using RNAi approach for the suppression of MIPS activity exhibited reduced apical dominance, precocious leaf senescence as well as a reduction in overall tuber field [27]. Interestingly, the phosphorus derived from phytic acid are important nutrient source contributing to the plants flowering [30,31]. Therefore, the vernalization induced a series of predominant physiological performances such as the early flowering and fast growth in plants probably depend on these crucial primary metabolites by the up-regulation of phytic acid biosynthesis pathway as our quantitative proteomic data revealed.

### The glycine-rich RNA binding proteins contribute to the vernalization-induced flowering process

The glycine-rich RNA binding proteins (GRPs) are composed of the RNA-recognition motifs at the N-terminus and a glycine-rich region at the C-terminus [32]. It was well known that the cellular transcript levels for GRPs in different plants are significantly increased by the out layer of stimuli such as cold, drought and pathogen infection [33–36]. Accordingly, a total of seven GRPs were identified and quantified with consistent increasing protein abundance in the vernalization-treated proteomes of faba bean (S2 Table). Such up-regulation of GRPs was also observed among other proteomic investigations on Arabidopsis and wheat after vernalization [5,13], suggesting an important and conserved role in plants response to vernalization. Interestingly, a number of RNA binding proteins act as suppressor of the *FLOWERING LOCUS C* (FLC) that is a flowering repressor and a crucial signaling element of vernalization response in Arabidopsis [8,37]. Meanwhile, the glycine-rich RNA binding protein AtGRP7 play an important role in promoting floral transition in Arabidopsis, further strongly indicating that the GRPs probably participate in flowering-time regulation response to the vernalization effects in faba bean.

### qRT-PCR expression analysis of selected genes with corresponding protein changes in faba bean upon the vernalization treatment

**Fig 6:**
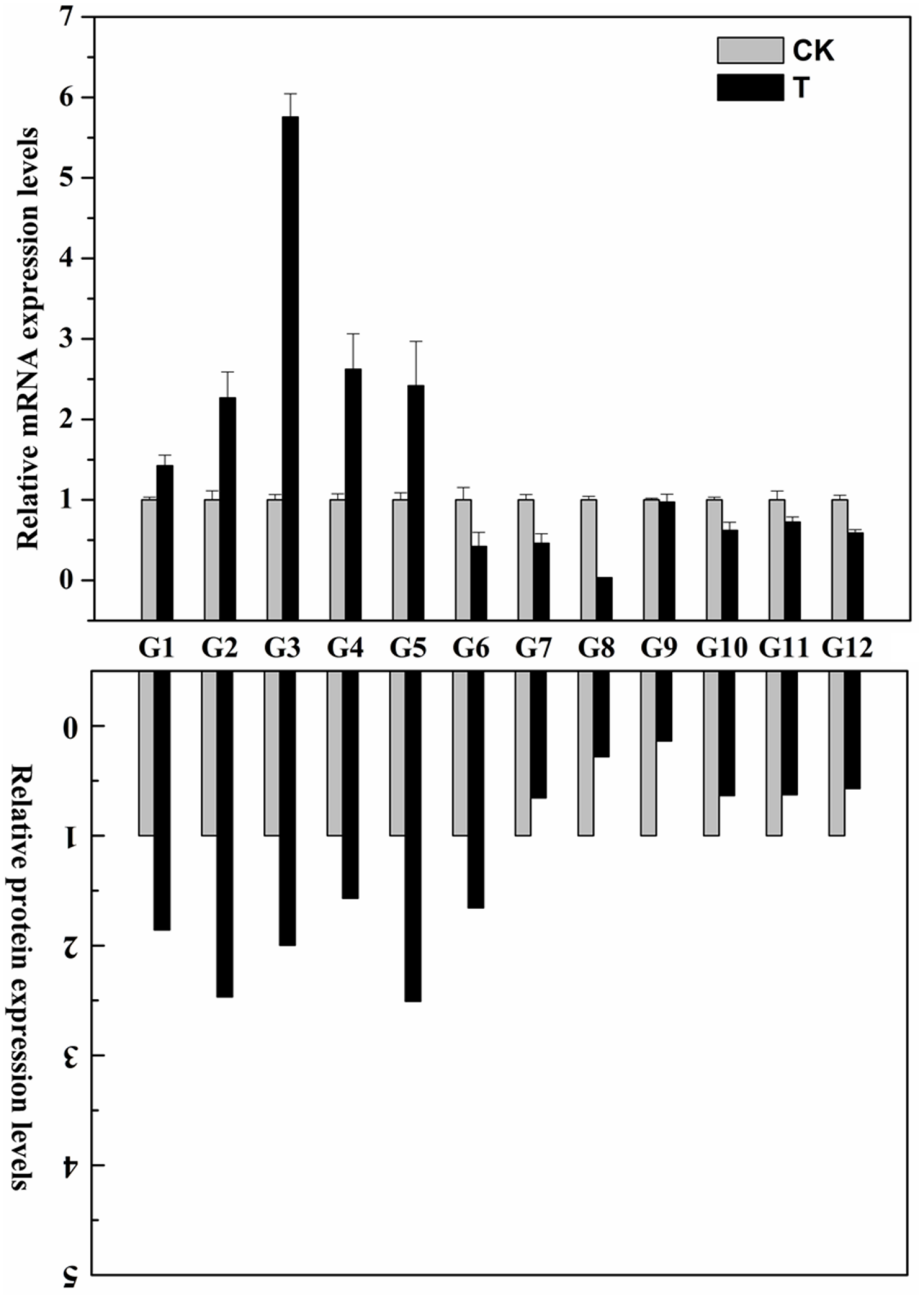
qRT-PCR expression analysis of selected genes with corresponding protein changes in Faba bean upon the vernalization treatment. Faba bean seedlings were analyzed following 18 days of vernalization treatments. Relative expression levels in vernalization-treated plants were normalized against the non-treated WT plants. Bars represent SE (n = 3); The relative protein expression levels of selected proteins were derived from proteomics data of this iTRAQ-based experiment. G1: VF_4569; G2: VF_1775; G3: VF_5337; G4: Uni_12222; G5: Uni_15562; G6: VF_9725; G7: Uni_10551; G8: VF_4918; G9: Uni_13160; G10: VF_6886; G11: Uni_1350; G12: VF_6654.

To determine whether the differentially expressed proteins are associated with transcriptional changes, qRT-PCR analysis was performed to detect the correlation between protein and gene expression in this study. As shown in Fig 6, approximately 80% of the selected genes showed down-regulated and up-regulated expression levels in Faba bean seedlings after the vernalization treatment, consistent with the changes in abundances of the corresponding proteins as revealed from the iTRAQ-based experiment (S1 Table), indicating a high congruency between protein and gene expression and a high quality of the quantification results in this study. Interestingly, the expression levels of VF_9725 and Uni_13160 were found to have down-regulated and no significant changes in the vernalization-treated plants compared to non-treated plants (Fig 6). However, increased abundance of VF_9725 and decreased abundance of Uni_13160 were detected in the vernalization-responsive proteome of this study. Therefore, the differential expression levels of their corresponding proteins are probably regulated by various post-transcriptional modification such as alternative splicing, RNA processing or other effects on translation efficiency [16], indicating the existence of a highly complex regulatory network in faba bean seedlings upon the vernalization treatment.

## Discussion

### Little changes have been detected in transcription factor family by iTRAQ-based quantitative proteomics

Transcriptome profiling detected substantial transcript changes in response to vernalization process [38,39]. Subsequently, transcription factors (TFs), a large group of proteins which are responsible for this transcriptional regulation have drawn much attention in last decade [40]. In higher plants, over 1,000 putative genes encoding transcription factors have been reported in each species [40], indicating the complexity of transcription regulatory hierarchy. Although many TFs have been documented to regulate flowering [41–43], few of them have been reported to participate in vernalization regulation. One example is the famous TF FLC has been demonstrated to induce flowering under vernalization by triggering systemic responses in Arabidopsis meristem [44]. In this study, two putative transcription factor encoding genes, VF_6231.Contig1_All and VF_5961.Contig2_All, have been short-listed as differential expressed proteins during vernalization. The VF_6231.Contig1_All encodes a protein which is homologous to rice BTF3 transcription factor. The down-regulation of this protein resulted in embryo lethal phenotype in rice, suggesting its role in plant development[45]. However, its role in vernalization remains to be elucidated. Another gene, VF_5961.Contig2_All, encodes a bZIP transcription factor with unknown function. Further physiological and biochemical studies are needed to unravel the molecular mechanisms of these TFs in regulating transcription networks during vernalization. Meanwhile, the presence of only two TFs in the list of differential expressed protein may also indicate that the regulations of these TFs are not confined to the protein level. Post-transcriptional regulations such as alternative splicing and post-translational regulations including phosphorylation and *N/O*-glycosylation may exist as well.

### RNA splicing may contribute to an extra layer of regulation during vernalization

In addition to 5’-capping and 3’-polyadenylation, RNA splicing is considered to be one of the most important post-transcriptional checkpoints. It is processed by a complex of proteins which are preferentially aggregated in spliceosome. Amongst these spliceosome-associated proteins, increasing attention has been drawn to a category of proteins named as splicing factors (SFs) [46]. Alternative splicing (AS) can generate multiple transcript variants from a single pre-mRNA, and it accounts for over 95% of intron-containing genes in mammals [47], which in turn to amplify the potential proteome diversity. Considerable evidence indicated that AS may regulate a brand new pathway other than conventional transcriptional control [46,48–50]. Furthermore, recent transcriptome profiling analysis indicated that approximately over 60% intron-containing genes undergo AS in several plant species including maize, Arabidopsis and soybean [44, 47], suggesting the similar AS regulation may exist in faba bean. In this study, a number of glycine-rich RNA-binding proteins have been observed to be up-regulated during vernalization, indicating the possibility that AS-regulated mechanism play an important role during this process. However, further investigation is needed to unravel the function of these proteins such as their target transcripts and RNA binding sites in faba bean.

### Omics approaches are powerful tools to resolve the molecular regulatory circuits of vernalization

Given that the complex nature of the vernalization process, utilization of a single proteomic approach is hard to fully understand the molecular profiles. Furthermore, labeling proteomics also share common drawbacks including insufficient label caused loss of detection, the complexity of PSM identification and relatively low reproducibility *etc*. Label-free method such as SWATH-MS based proteomics may increase the number of identified differential expressed proteins and the reproducibility at the same time [51,52]. In addition, the proteomic methods are limited by their low coverage and throughput, which is can be complemented by transcriptome analysis such as the next-generation of RNA sequencing. Using proteogenomics approach [53], an combined analytical flow that can analyze transcriptomic and proteomic data simultaneously, maybe more informative to look at the alternation of both transcripts and proteins. Definitely, functional characterization is required to validate the findings from bioinformatic analysis. The combinatory approaches will deeper our insights of vernalization process in faba bean.

## Conclusion

There already have sufficient proteomics investigations on the proteome changes in a variety of plant species responding to diverse biological and environmental cues. However, the proteome information on vernalization response is rarely reported and only limited research have been investigated on Arabidopsis and wheat through the traditional gel-based proteomics methods [5,13]. Our study employs the iTRAQ-LC-MS/MS platform to accurately identify and quantify proteome changes in faba bean upon vernalization treatment. Evaluation of differentially expressed protein profiling indicated several potential regulators of vernalization responses in faba bean. Down acuumulation of photosynthesis-related proteins during vernalization, increased phytic acid biosynthesis, which would provide protein evidence that how faba bean regulate its biological process during vernalization. In addition, the accumulation of several glycine-rich RNA-binding proteins after vernalization may also shed light on the potential AS regulation on this process. Therefore, our iTRAQ-based quantitative proteomic profiling in combination with further molecular and genetic characterization would offer deep insights into the regulatory mechanism of vernalization and pinpoint major regulators for potential agricultural applications in future.

## Supporting information

S1 Table List of proteins that respond to vernalization in Faba bean seedlings. (XLSX)

S2 Table Full identification and quantification of protein list. (XLSX)

S1 Fig Quality control validation of MS data. (A) Mass error distribution of all identified peptides. The distribution of mass error is near zero and most of them are less than 0.02 which means the mass accuracy of the MS data fit the requirement. (B) Peptide length distribution. The length of most peptides distributed between 8 and 16, which agree with the property of tryptic peptides.
(TIF)

S2 Fig Reproducibility analysis of three repeated trials by Pearson correlation coefficient. (TIF)

S3 Fig GO enrichment-based clustering analysis. (A) Distribution of quantification results. (B) GO enrichment-based clustering analysis of differentially expressed proteins.
(TIF)

S4 Fig Protein domain and KEGG pathway enrichment-based clustering analysis. (TIF)
