# Supplementary figures and images for: iTRAQ-based quantitative proteomic analysis in vernalization-treated faba bean (Vicia faba L.)
Source: PLoS One. 2017 Nov 9;12(11):e0187436. doi: 10.1371/journal.pone.0187436 (PMC5679601; doi:10.1371/journal.pone.0187436)

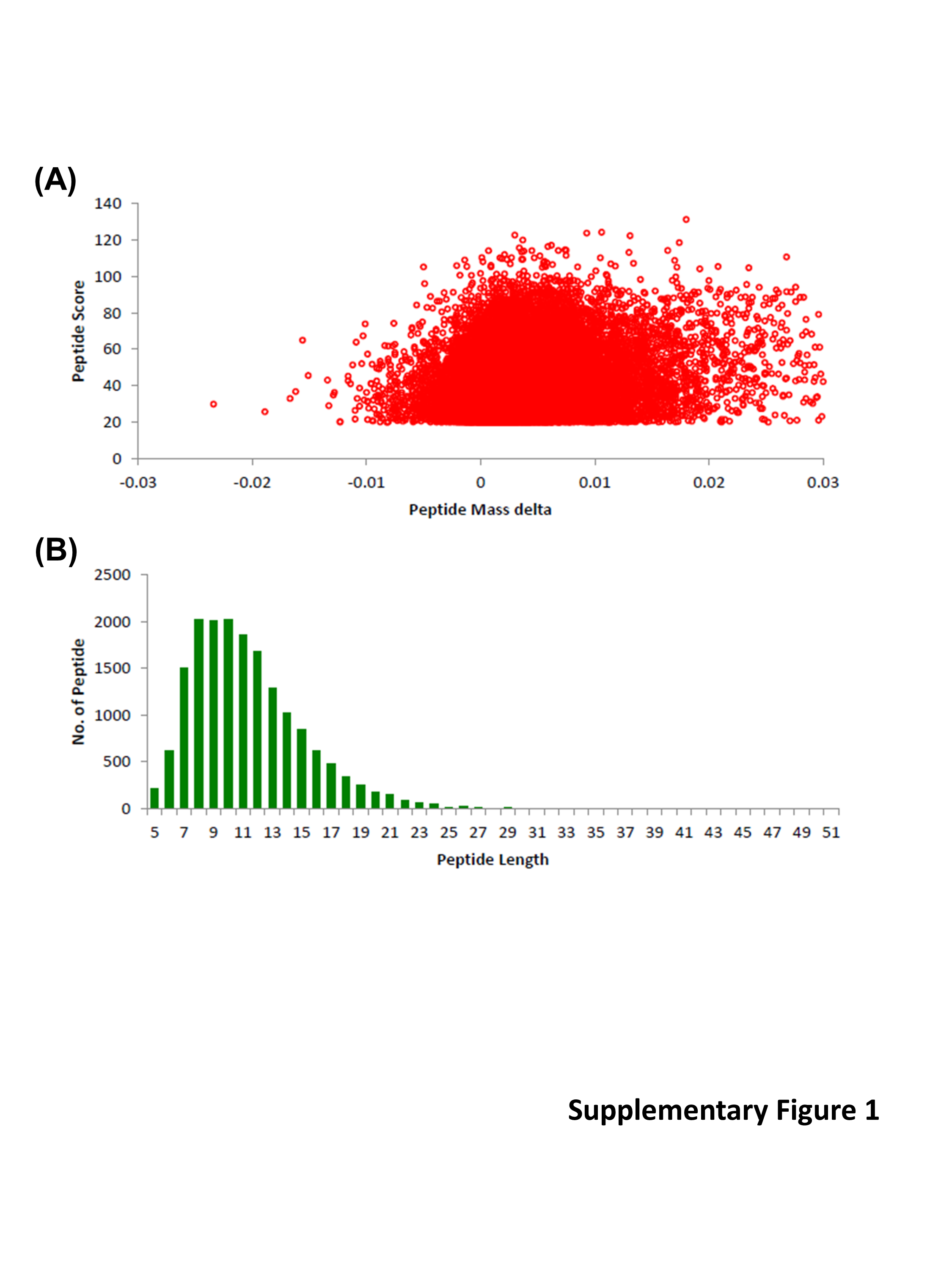

Supplement: S1 Fig — (A) Mass error distribution of all identified peptides. The distribution of mass error is near zero and most of them are less than 0.02 which means the mass accuracy of the MS data fit the requirement. (B) Peptide length distribution. The length of most peptides distributed between 8 and 16, which agree with the property of tryptic peptides. (TIF) [file pone.0187436.s003.tif]

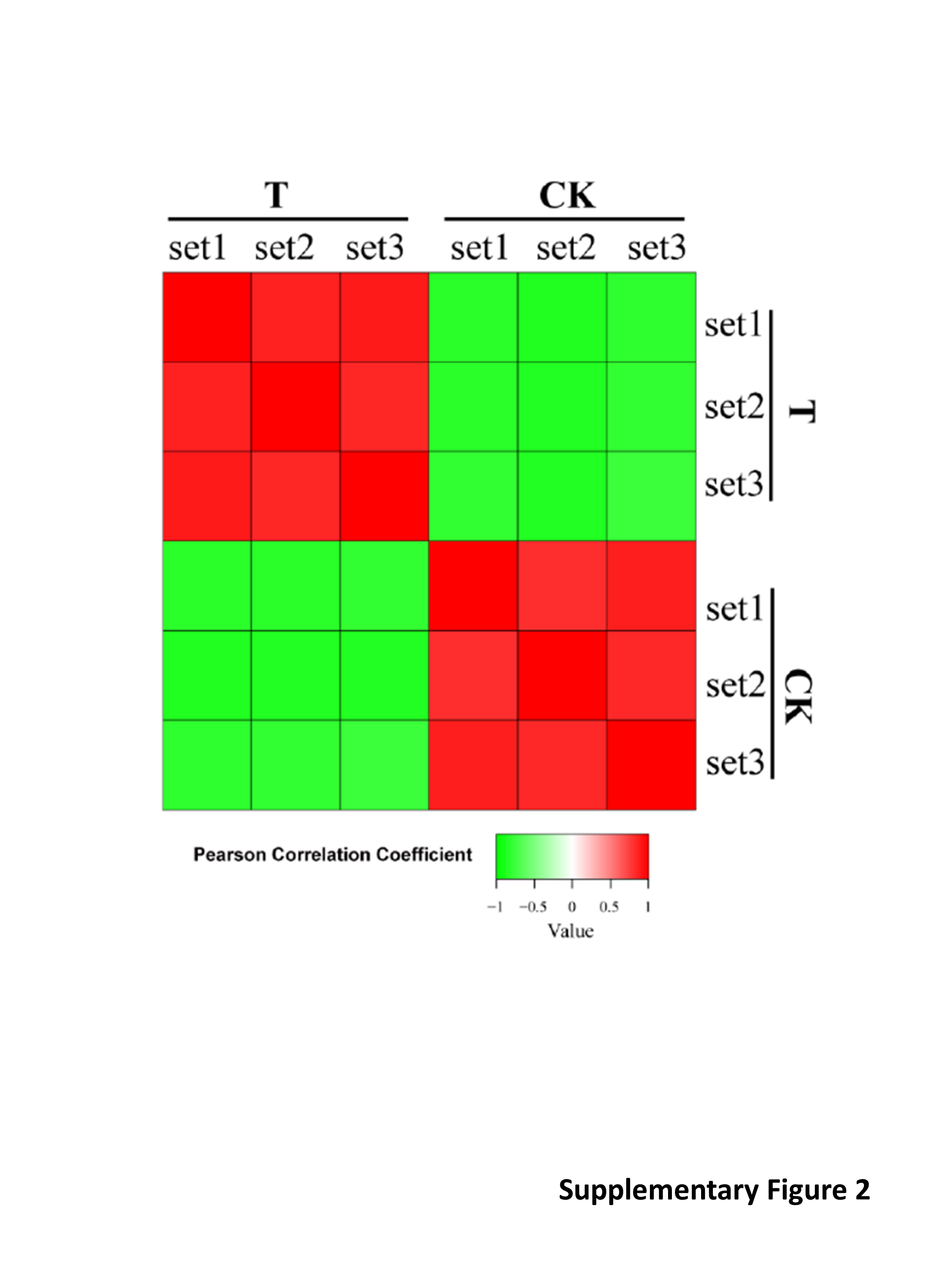

Supplement: S2 Fig — (TIF) [file pone.0187436.s004.tif]

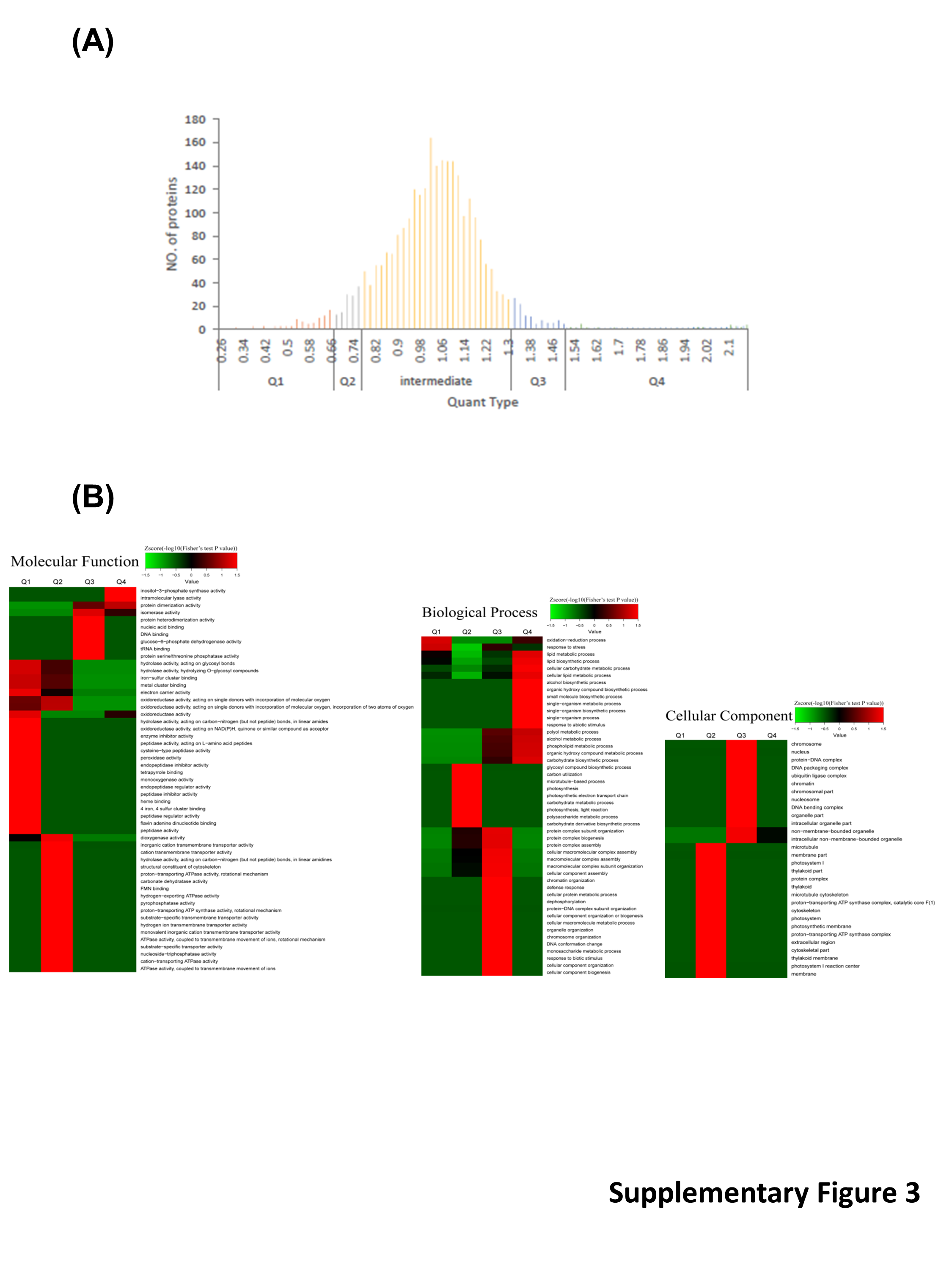

Supplement: S3 Fig — (A) Distribution of quantification results. (B) GO enrichment-based clustering analysis of differentially expressed proteins. (TIF) [file pone.0187436.s005.tif]

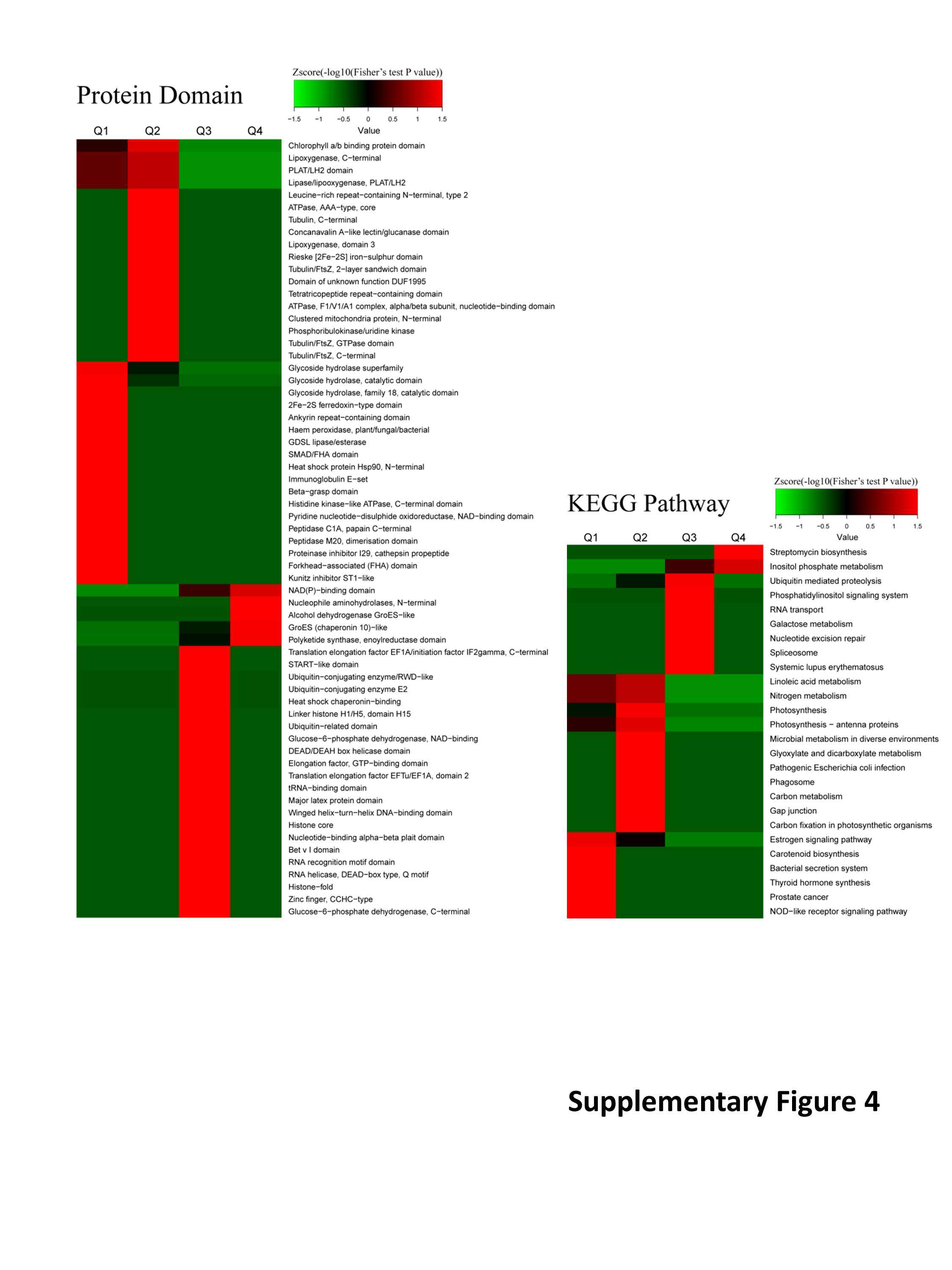

Supplement: S4 Fig — (TIF) [file pone.0187436.s006.tif]
